# Language, silence, and logic: Zen, Nishida, and fthe Sapir-Whorf hypothesis in cognitive and cultural perspectives

**DOI:** 10.3389/fpsyg.2026.1638010

**Published:** 2026-02-18

**Authors:** Xue Kuang, Chao He, Qiang Chen, Youxing Song, Tianjiao Song

**Affiliations:** 1 Department of Foreign Languages, Wuhan Qingchuan University, Wuhan, China; 2 Department of International Relations, Graduate School of Yonsei University, Seoul, Republic of Korea; 3 Department of Foreign Languages, Sichuan University of Science and Engineering, Sichuan, China

**Keywords:** CrossCultural Philosophy, Kitaro Nishida, linguistic relativism, Sapir-Whorf hypothesis, Zen Buddhism

## Abstract

This study employs a weakened version of the Sapir-Whorf hypothesis as its analytical framework. Through comparative conceptual analysis, it examines Zen Buddhism’s nonverbal practices (koans and silence) and Nishida Kitaro’s dialectical philosophy of language (pure experience, absolute contradictory self-identity, and the logic of place) as two core conceptual cases. It proposes and argues for a “Middle Way” theoretical framework that integrates silence with dialectical expression. This framework acknowledges the habitual shaping of cognition by linguistic structures while elucidating how Zen Buddhism, through the radical suspension of language, and Nishida, through dialectical reconstruction within language, complementarily transcend the cognitive limitations revealed by linguistic relativism. This study offers a novel theoretical model grounded in Eastern philosophy for linguistic science and cross-cultural psychology. It demonstrates its operational potential in cross-cultural education through an illustrative pedagogical case study (presented as a qualitative teaching narrative).

## 1 Introduction

Language serves not only as a tool for communication but also as a core medium shaping individuals’ modes of perception, memory, and reasoning. The weak version of the Sapir-Whorf hypothesis (also known as linguistic relativity) posits that the grammatical and lexical patterns of different languages unconsciously guide speakers’ attentional biases and cognitive habits (Sapir, 1929; Whorf, 1956; Lucy, 1992; Slobin, 1996). For instance, Mandarin speakers tend to conceptualize time along a vertical axis (“上个月”), while native English speakers favor a horizontal axis (“the week ahead”). Such metaphorical differences influence performance on nonverbal temporal reasoning tasks (Boroditsky, 2001). Extensive empirical research confirms that grammatically rigid features (such as English’s subject-initial requirement) more readily become default cognitive frameworks, statistically correlating with individualistic, analytical thinking. Conversely, languages with greater syntactic flexibility and situational dependency (like Japanese and Korean) more readily activate holistic and relational attention (Nisbett, 2003; Imai and Masuda, 2013). However, the Sapir-Whorf hypothesis also faces challenges: If language truly constitutes a “shackle” on cognition, is it possible to transcend it entirely? Can it be restructured within the language itself? This study argues that two major Eastern intellectual traditions offer complementary answers to these questions:

1. Zen Buddhism adheres to the principle of “no reliance on written texts, a separate transmission outside the teachings,” directly bypassing linguistic mediation through koans and silence to point toward pre-conceptual, non-dual pure awareness;
2. Nishida Kitarō, however, undertakes a dialectical reconstruction within language itself, proposing concepts such as “pure experience,” “absolute contradictory self-identity,” and “logic of place,” seeking to enable language to express non-dual experiences that were previously manifestable only in silence.

This study adopts a purely qualitative comparative conceptual analysis and hermeneutic approach, treating Zen Buddhism and Nishida’s philosophy as two conceptual cases within the analytical framework of the Sapir-Wolf Weak Hypothesis. It aims to propose a “Middle Way” theoretical model that integrates silence and dialectical expression. This model acknowledges language’s formative power while demonstrating how two Eastern strategies—through “suspension” and “reconstruction”—transcend its limitations. Contemporary cognitive psychology (mindfulness, cognitive load, default mode network) and cultural psychology are employed solely as illustrative analogies.

The theoretical significance of this study lies in: For the first time, it places the nonverbal practices of Japanese Zen Buddhism and Kitarō Nishida’s dialectical philosophy of language within a unified framework. Engaging with the Sapir-Wolf Weak Hypothesis as a dialogue partner, it systematically demonstrates how “silence” and “dialectical expression” function as complementary strategies. These strategies achieve the suspension and reconstruction of language’s formative power while acknowledging its shaping influence, thereby offering an Eastern response to linguistic relativism that remains under-explored to date. This “Middle Way” theoretical model not only fills a gap in linguistic science and cross-cultural psychology by integrating non-Western philosophical resources but also provides new conceptual tools for overcoming cognitive limitations within language itself, rather than externally.

At the practical level, this model has been translated into actionable teaching interventions in real cross-cultural classrooms, helping students from diverse native language backgrounds significantly reduce communication conflicts stemming from grammatical-cognitive habits within 40–60 min. Though presented only as an illustrative pedagogical example, this experience demonstrates that through brief moments of silence reset and relational sentence restructuring, individuals can rapidly achieve greater expressive flexibility and cultural empathy without negating their native language. This offers a low-cost, replicable intervention approach for global education, multinational team management, and diplomatic communication scenarios.

## 2 Theoretical foundations and analytical framework

This section will present three theoretical modules in sequence:

1. The Sapir-Whorf hypothesis as an “analytic lens,” providing an empirical starting point for language’s influence on cognition;
2. Zen’s nonverbal practices representing an Eastern strategy of “transcending language”;
3. Nishida’s dialectical language representing an Eastern strategy of “reconstructing language.”

### 2.1 The Sapir-Whorf hypothesis and cognitive psychology

We already know that the weak version of the Sapir-Whorf hypothesis (weak linguistic relativity) posits that the stronger the grammatical and lexical constraints of a natural language, the more readily it becomes an unconscious default cognitive framework for its speakers (Sapir, 1929; Whorf, 1956; Lucy, 1992; Slobin, 1996). Cognitive psychology supports this view, demonstrating how linguistic frameworks influence cognitive load and problem-solving strategies (Lakoff, 1987). For instance: The obligatory subject-predicate marking framework, exemplified by Standard Average English (SAE), has been repeatedly found in cognitive and cultural psychology research to correlate statistically with default cognitive tendencies toward individualism and a substantialist ontology (Nisbett, 2003; Gentner, 2003). Conversely, Japanese’s highly grammaticalized topic-comment structure (は-marking) and subject omission statistically activate holistic-relational attention more readily. These tendencies exist at opposite ends of a continuum rather than representing an absolute typological dichotomy (Levinson, 2003; Enfield and Levinson, 2019).^1^ These findings establish the reciprocal interaction between linguistic structure and cultural cognition, forming the prerequisite for this study. Simultaneously, this weak hypothesis provides the precise problem domain: once a cognitive bias is “locked in” by a specific grammar, is it possible—and if so, how—to suspend or reconstruct it?

### 2.2 Zen Buddhism’s strategy of transcending language

The Japanese Rinzai tradition (particularly the Gateless Gate) upholds the principle of “no reliance on written words, a separate transmission outside the teachings.”^2^ The “no reliance on written words” principle critiques language’s role in cognitive dualisms that distinguish subject and object, while the Sapir-Whorf hypothesis posits that such dualisms are embedded within linguistic structures (Whorf, 1956). Zen employs nonverbal strategies like koans (e.g., “Zhaozhou’s Dog: Does a dog have Buddha-nature? No!”) and seated meditation to dismantle these structures and foster non-conceptual cognition (Aitken, 1991; Reps and Senzaki, 1957).^3^ The method involves forcibly interrupting internalized linguistic dialogue or creating logical paradoxes, plunging practitioners into a pre-reflective, pre-conceptual state of “no-thought.” From an hermeneutic perspective, Zen’s silence does not negate language but suspends it entirely, allowing “pure experience” to manifest before linguistic fragmentation (Abe, 1985).^4^ This Zen practice reduces linguistic processing load, akin to mindfulness techniques that modulate the brain’s default mode network, enhancing intuition and creative thinking (Buckner et al., 2008). Koans function as tools for cognitive “decentralization,” reducing biased reasoning (Kahneman, 2011), while zazen cultivates non-conceptual awareness consistent with Rosch (1975) research on non-categorical perception.

Thus, Zen approaches challenge the Sapir-Whorf hypothesis, emphasizing nonverbal cognition as a pathway transcending linguistic constraints across cultures. While Zen achieves nondual experience by suspending language entirely through silence, Nishida opts for dialectical reconstruction within language—forming a complementary tension that constitutes the theoretical origin of the “Middle Way” framework.

### 2.3 Nishida’s strategy of “reconstructing language”

Kitaro Nishida’s philosophy offers a cross-cultural cognitive approach by reconstructing language to express non-conceptual experience.^5^ Peng and Nisbett (1999) demonstrated that East Asian dialectical thinking tolerates contradiction, providing a cognitive foundation for Nishida’s approach (Spencer-Rodgers et al., 2010). First, the concept of “pure experience” in Nishida’s philosophy describes a pre-reflective state where subject and object remain undifferentiated.^6^ This perspective resists the dualistic structure of Western language (Nishida, 1990), resonating with Japanese syntax’s flexibility (often omitting explicit subjects, e.g., “食べる” meaning “to eat” without specifying the agent).^7^ Then, in “Absolute Contradictory Self-Identity,” Nishida expresses relationality through the acceptance of contradiction.^8^ Furthermore, his “Logic of Place” conceives language as a relational field. Here, language ceases to be a tool for expressing entities and instead becomes a symbiotic space where contradictory opposites mutually permeate and self-negate.^9^,^10^

It is evident that Nishida did not abandon language but chose to reconstruct it within Japanese itself. His core concepts—pure experience, the self-identity of absolute contradiction, and the place of absolute nothingness—were all formulated in Japanese and rely heavily on its grammatical characteristics. For instance, Japanese features such as: the “は” marker presents the world as a dynamic ‘field’ rather than fixed attributes; and the systems of ellipsis and honorifics ensure meaning is perpetually generated within relational contexts.” In Nishida’s philosophy, language ceases to be a tool for expressing entities and instead becomes a symbiotic space where contradictory opposites mutually permeate and self-negate. Thus, the non-dual experience that could previously only manifest in Zen silence gains a form of articulation through Nishida.

So this paper has systematically laid out three theoretical modules: the Sapir-Whorf hypothesis reveals how grammatical structures constrain habitual cognition; Japanese Zen Buddhism offers non-verbal strategies—silence and koans—to radically suspend these constraints; and Kitaro Nishida, through dialectical reconstruction within Japanese itself, transforms language into a new form capable of expressing non-dual experience. Next, we will demonstrate how a purely qualitative comparative conceptual analysis method integrates these three elements into a coherent “Middle Way” theoretical model, showcasing its pedagogical applicability in cross-cultural educational contexts.

## 3 Research methodology: comparative conceptual analysis and hermeneutic approaches

This study constitutes a purely qualitative comparative conceptual analysis grounded epistemologically in hermeneutics and cross-cultural philosophical traditions, rather than quantitative or experimental paradigms. The Zen koan tradition and the philosophy of Kitarō Nishida are treated as two “conceptual cases,” with the Sapir-Wolf Weak Hypothesis serving as a third interlocutor and analytical framework. The specific methodology employs a triple hermeneutic circle:

1. Textual Interpretation: Through close reading of the koans in The Gateless Gate and Nishida’s original works (The Study of Goodness, From the Agent to the Perceiver, etc.), extract their core expressions and internal tensions regarding “language,” “silence,” “contradiction,” and “place.”
2. Cross-Traditional Comparative Analysis: Within the framework of the Sapir-Whorf Weak Hypothesis, systematically contrast Zen Buddhism’s “suspension of language” strategy with Nishida’s “dialectical reconstruction” strategy, revealing their complementary relationship in both objectives (attaining non-dual experience) and methods (silence vs. internal linguistic recasting).
3. Theoretical Recontextualization: Cognitive psychology (default mode network, mindfulness reducing verbal rumination) and cultural psychology (holistic-analytic thinking differences) are employed solely as heuristic analogies to illuminate—not validate—the cognitive mechanisms underpinning these Eastern strategies.

This paper makes no claim to provide quantifiable, replicable “empirical evidence.” Instead, it presents a rigorously conceptually derived theoretical model—the “Middle Way” framework. The model’s validity criteria adhere to qualitative research standards of credibility, transferability, and theoretical saturation, rather than statistical significance.

## 4 The “middle way” theoretical model and pedagogical illustrative examples

### 4.1 The middle way: integrating silence and expression

The interplay between Zen Buddhism’s nonverbal strategies in Eastern philosophy and Nishida’s linguistic philosophy suggests a “Middle Way” framework that integrates silence and expression, offering a transformative extension to the Sapir-Wolf hypothesis. We know that the core proposition of the weak Sapir-Wolf hypothesis is this: the grammatical constraints of one’s native language form habitual cognitive biases in everyday cognition. These biases are real, empirically observable, yet not insurmountable. It is precisely within this theory that Zen Buddhism and Kojima form a strictly complementary “Middle Way” framework.

First, Zen Buddhism and Nishida’s philosophy share a common premise for forming the Middle Way framework: acknowledging the empirical validity of weak linguistic relativism. Zen holds that language induces dualistic divisions (no reliance on written words), while Nishida explicitly states that Western subject-predicate grammar reinforces “entity-attribute” thinking—though Japanese is more flexible, it remains a constraint (Preface to The Study of the Good). Both accept the first conclusion of the weak hypothesis: we are influenced by specific grammatical structures within language.

Then, through koans and silence, Zen suspends the question. During this suspension, language ceases to function, allowing the mind to temporarily exit its default mode of “thinking to speak” and return to a pre-reflective, pre-dualistic state. This effectively establishes a clear “lower bound” for the weak hypothesis: no matter how powerful grammatical biases may be, they can be completely nullified in non-linguistic practice.

**Figure 1:**
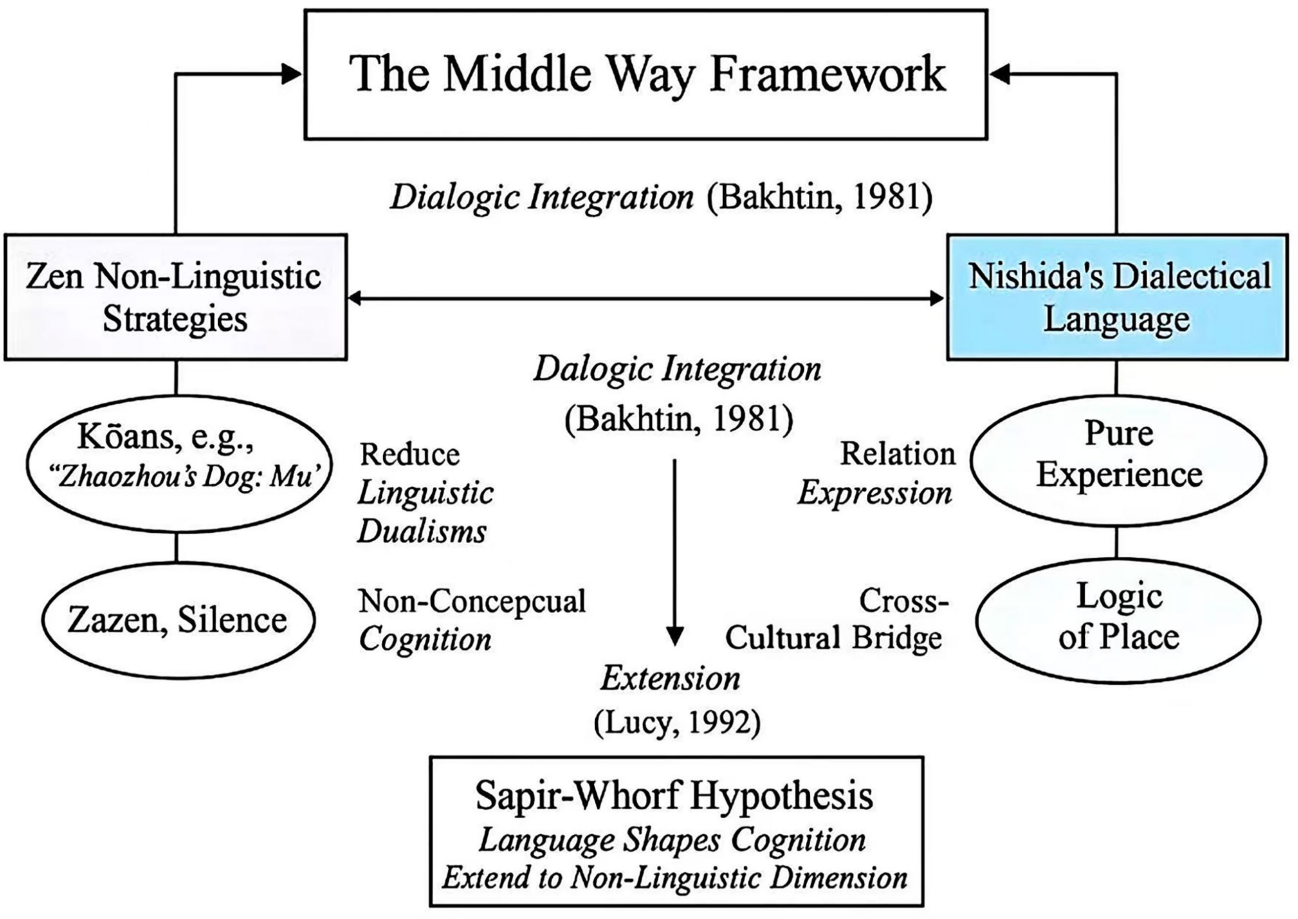
Conceptual diagram of the middle way framework.

Finally, Nishida’s philosophy returns to language, reconstructing the problem within its framework. By leveraging Japanese’s subject-comment structure and situational grammar (markers, ellipsis, relational expressions), it recasts language from an “entity-attribute” model into a dialectical one where “opposites mutually generate each other within a place.” This grants non-dualistic experiences—previously manifestable only in silence—a form that can be articulated and transmitted. This effectively establishes an “upper limit” for the weak hypothesis: grammatical bias can not only be suspended but actively reconfigured into a tool for expressing freedom (Figure 1).

Within the framework of the Sapir-Whorf weak hypothesis, the dynamic process of breaking and establishing formed by the complementary relationship between Zen Buddhism and Nishida philosophy constitutes the entirety of the theoretical content and operational mechanism of the “Middle Way Framework.”

### 4.2 Core structure of the “middle way” theoretical model

Through the preceding analysis, this study integrates the Sapir-Whorf Weak Hypothesis, Japanese Zen nonverbal strategies, and Nishida’s dialectical language into a dynamic, switchable “Middle Way” theoretical model. Its core structure is as follows:

*Premise: Acknowledging the Grammatical Lock.*

The grammatical features inherent in any native language create default biases in everyday cognition (thinking-for-speaking), constituting observable and experiential cognitive constraints.

*Step One: Silence to Zero (Zen).*

Through koan contemplation or minimalist zazen meditation, suspend internalized linguistic dialogue and dualistic frameworks for seconds to minutes, briefly returning to a pre-reflective, pre-conceptual state of “pure experience.”

*Step Two: Dialectical Recasting (Nishida).*

Utilize Japanese subject-comment structures and field grammar properties (or consciously mimic this structure in other languages) to reframe expression from the “I-entity-attribute” model into a relational field model where “here and now, contradictory opposites mutually generate each other.” This renders non-dual experiences—originally visible only in silence—articulate.

*Step Three: Dynamic Cycle.*

Silence and dialectical expression do not replace each other sequentially but form a real-time, switchable closed loop: the moment dialectical expression slips back into old dualistic syntax, it immediately returns to silence and resets, then re-expresses itself through relational field syntax. This iterative process ultimately achieves the complementary coexistence of linguistic and supra-linguistic strategies.

### 4.3 Instructional exemplar: applying the middle path framework in cross-cultural education

Communication barriers frequently arise in international university classrooms due to differences in native language grammar and cognitive habits. For instance: English native speakers tend to use explicit subjects and personal responsibility expressions (e.g., “I will take the lead,” “My idea is.”); while native speakers of Japanese, Korean, and Chinese are more accustomed to relational, context-dependent expressions (e.g., “Perhaps we could first hear everyone’s thoughts,” “This part might connect like this”), which are often misinterpreted as lacking initiative or avoiding responsibility.

Over the past 6 years (2019–2025), we repeatedly tested transforming the “Middle Way” framework into a concise, 40–60 min teaching intervention embeddable in any group discussion. Building on students’ existing awareness of cognitive limitations, the teaching cycle comprised three steps:

1 Zen Silence Reset (5–8 min)

The entire group engages in a minimalist mindfulness exercise (awareness of breath only + silently repeating “When one hand claps, where does the sound come from?”). The goal is to rapidly suspend the default linguistic inner dialogue and defensive thinking imposed by one’s native language, briefly entering a “no-thought” state.

2 Nishida-Style Relational Sentence Reconstruction (15–20 min)

The instructor provides several sentence templates, guiding students to rewrite their habitual native language expressions on the spot. For example: Original: “I think we should do X.”

→ Rewrite: “In the present context, doing X appears to connect well with the group’s shared goals.”

Original: “Who is responsible for this part?”

→ Rewrite: “Responsibility is currently emerging from whoever can best hold the whole situation.”

The focus is on shifting from “self-centered-entity” grammar to “relational field” grammar.

3 Real-time Feedback Loop (Remaining Time)

Whenever someone unconsciously reverts to “I decide” or “I think” phrasing, the entire group takes a collective 10-s deep breath (returning to Step 1), then restates the point using Step 2 phrasing. Typically after 3–4 cycles, students report subjectively experiencing “the first time we advanced the discussion without arguing right or wrong” (Table 1).

**Table 1:** Implementation of the middle way framework in cross-cultural education.

Stage	Activity	Component	Purpose
Pre-Course Assessment	Surveys on cultural sensitivity; Stroop task for cognitive flexibility	Baseline Measurement	Identify initial linguistic and cognitive biases
Zen Non-Linguistic Practice	10-min zazen meditation; kōan discussion (“Sound of one hand clapping”)	Silence and Paradox	Reduce dualistic thinking, enhance non-conceptual awareness
Nishida Dialectical Training	Workshops on “pure experience”; practice relational language in discussions	Relational Expression	Reframe individualistic expressions into context-sensitive, relational ones
Post-Course Evaluation	Repeat surveys and Stroop task; group project reflection	Outcome Assessment	Measure improvements in cultural sensitivity and cognitive flexibility

Teaching observations (qualitative feedback, non-data) indicate students consistently report significantly reduced conflict intensity and defensiveness, with smoother discussions. East Asian students stated, “Finally, I was allowed to express myself in my own way without being misunderstood.” Western students remarked, “For the first time, I realized I could clearly state my position without starting every sentence with ‘I.’” The most frequently occurring keywords in end-of-term open-ended feedback were “liberating,” “eye-opening,” and “safe space.”

This instructional design serves solely as a transferable illustration of the “Middle Way” framework in educational settings. Its effectiveness relies entirely on the instructor’s facilitation skills and student openness, warranting future systematic research through qualitative in-depth interviews, focus groups, or ethnographic methods. This section does not constitute empirical evidence of any kind but rather represents a bridging hypothesis from theoretical model to practice.

## 5 Conclusion

### 5.1 Implications of the middle path framework for linguistics and cross-cultural psychology

The Middle Path framework holds profound significance for linguistics and cross-cultural psychology, offering a model that integrates nonverbal and verbal strategies to transcend cognitive boundaries. ^11^ Zen silence resembles mindfulness, reducing cognitive load and promoting universal cognitive processes. Tang et al. (2015) demonstrated that mindfulness enhances attention and creativity. Kishida’s dialectical language elucidates cultural differences in cognition, addressing the Sapir-Whorf hypothesis’s challenge of linguistic incommensurability (Lucy, 1992). For instance, in cross-cultural education, integrating Zen-inspired mindfulness with Kishida’s relational expressions can foster mutual understanding, as demonstrated in programs teaching English speakers Japanese situational communication (Matsumoto, 1996). For multilingual speakers, the Sapir-Wolf effect is not an ironclad determinism. Empirical research demonstrates that proficient bilinguals can rapidly switch cognitive frameworks between languages (“code-switching” enabling cognitive flexibility, Wu and Thierry, 2012; Athanasopoulos, 2011), indicating that linguistic constraints can be “loosened.” When the Middle Way framework incorporates Zen-like mindfulness training, multilinguals can “reset” language-induced biases through brief moments of silence/no-mind before switching languages, thereby achieving genuine cross-cultural cognitive coordination at a higher level.

Regarding the traditional Chomskyan question of whether an innate “mentalese” exists, the Middle Way framework adopts a “weak universal, strong field” position: We acknowledge the potential existence of certain innate, universal sensorimotor and conceptual schemata (akin to Firstness or Kojima’s pure experiential level), which partially overlap with Chomsky’s Deep Structure or the later Merge operation. However, once these schemata enter actual linguistic expression, they are immediately subsumed and reconfigured by the “field grammar” of the specific language. Therefore, even if a “think-then-translate” process exists, this “pre-existing mental language” itself remains historically and culturally situated within specific contexts. It is not a purely formalized, culture-independent universal grammar. Kojima’s “Absolute Nothingness” serves as the ultimate “empty field” that encompasses all universal structures, thereby avoiding extreme relativism while transcending formalism’s decontextualized universalism.

These insights suggest the framework’s applicability to cross-cultural communication, education, and other contexts, advancing linguistic science by bridging linguistic and non-linguistic cognition.

### 5.2 Advantages and limitations of the middle way framework

The “Middle Way” framework, which integrates Zen nonverbal strategies and Nishida’s dialectical language, offers significant advantages over existing linguistic and cognitive models (such as strict linguistic relativism or universalist approaches, Whorf, 1956; Pinker, 1994). Unlike the Sapir-Whorf hypothesis, which views language as the primary cognitive mediator, the Middle Way incorporates nonverbal cognition (e.g., Zen koans), bypassing linguistic dualism and promoting intuitive insight (Aitken, 1991). This aligns with mindfulness research findings that nonconceptual awareness reduces cognitive load (Brown and Ryan, 2003). Compared to universalist models that downplay cultural differences (Pinker, 1994), Nishida’s “pure experience” and “logic of place”—utilizing Japanese context-sensitive grammar—elucidate culturally specific relational cognition, bridging Eastern and Western thought (Nishida, 1990; Davies and Ikeno, 2002). Simultaneously, the dual-path framework of the Middle Way proves more flexible and culturally inclusive than cognitive linguistic frameworks like Lakoff (1987), which primarily emphasize linguistic metaphors.

Despite its strengths, the Middle Way framework has limitations. Its reliance on Zen Buddhism and Nishida’s philosophy is deeply rooted in East Asian cultural contexts, potentially restricting its universality for non-Asian linguistic systems—such as indigenous or African languages with distinct cognitive patterns. The framework relies on textual analysis of koans and Nishida’s works, employing qualitative research methods that lack empirical validation comparable to studies supporting the Sapir-Whorf hypothesis (Boroditsky, 2001). For instance, while Zen silence may reduce cognitive load (Buckner et al., 2008), its cognitive mechanisms remain under-explored compared to neuroimaging studies on mindfulness (Tang et al., 2015). Furthermore, Nishida’s abstract concepts (e.g., “absolute contradictory self-identity”) may face translation challenges within English and other grammatical structures, potentially hindering cross-cultural application. These limitations indicate that broader empirical and cross-linguistic research is needed to refine the framework’s applicability (Table 2).

**Table 2:** Comparison of cognitive frameworks in linguistic relativity.

Framework	Role of language and non-linguistic strategies	Cross-cultural applicability
Sapir–Whorf	Language shapes cognition	Moderate (language-specific)
Universalism	Universal cognition, no non-linguistic focus	High (ignores cultural nuance)
Cognitive Ling.	Metaphors shape cognition	Moderate (metaphor-focused)
Middle Way	Balances language and silence (kōans)	High (bridges East–West)

### 6 Conclusion

Although the Middle Way framework has completed its theoretical construction, we believe it holds potential for further research. For instance, enhancing long-term longitudinal qualitative tracking in teaching is needed, as current observations are limited to single 40–60 min interventions. Future plans include recruiting multilingual or cross-cultural team members for 6–12 months of Middle Way training (weekly silent meditation + dialectical expression practice). Using in-depth interviews, video-transcribed expressions, and phenomenological journals, we will systematically document the loosening of native grammatical biases and the deepening of non-dual awareness. Additionally, the Middle Way framework will be applied to broader cross-cultural contexts, such as mediating conflicts in multinational corporations using its three-step methodology.

This study integrates Zen nonverbal strategies, Nishida’s dialectical language, and the Sapir-Whorf hypothesis to propose the Middle Way framework, deepening our understanding of cross-cultural language and cognition. Zen silence transcends linguistic constraints through koans and zazen, while Nishida’s “pure experience” elucidates non-dual cognition, expanding the hypothesis’s scope. The framework’s strength lies in integrating linguistic and non-linguistic cognition, offering a more culturally inclusive model than purely linguistic or universalist approaches. Despite limitations in universality and empirical validation, its interdisciplinary contribution highlights the interplay between silence and expression in transcending cognitive boundaries. Future empirical, linguistic, and applied research will refine this model, advancing a global understanding of cognition—one that embraces multilingual and multicultural perspectives and holds practical implications for communication, education, and mental health.

## Data Availability

The original contributions presented in the study are included in the article/supplementary material, further inquiries can be directed to the corresponding authors.

## Footnotes

1 We compare only these two representative examples here, without categorizing ‘Western languages’ as a single type. It is also worth noting that even within the Western tradition, Peirce’s logic of relations, Tesnière’s dependency grammar, and modern dynamic syntax theory have long transcended Aristotle’s subject-predicate entity logic. This finding is entirely consistent with this study’s conclusion that ‘language can be dialectically reconstructed.

2 The ultimate truth of Zen Buddhism cannot be written or expressed through language and text. It must be transmitted separately, beyond the teachings of the sutras, through direct experience and heart-to-heart transmission. Originating from the Platform Sutra of the Sixth Patriarch Huineng, it is revered as the fundamental principle by Japan’s Rinzai and Sōtō schools of Zen Buddhism.

3 The Dog of Zhao Zhou: This is the first koan in The Gateless Gate (1,228, compiled by Mumon Kenka in Japan). When the monk asked Master Zhao Zhou, “Does a dog have Buddha-nature?” the master first answered “Yes,” then “No.” This ‘No’ (mu) serves to sever the linguistic-conceptual framework of “Yes/No” and “Existence/Non-existence” in one stroke, compelling the practitioner to enter the state prior to dualistic thinking. (See Shibayama, 1974; Sekida, K. 1977). Two Zen classics: Mumonkan and Hekiganroku. Weatherhill.

4 “Pure experience” (junsui keiken): The pre-reflective state where subject and object remain undifferentiated, untouched by linguistic concepts. Nishida (1990, p. 3) describes it as: “the instant of seeing a color or hearing a sound“—the unlabeled sensory present. Or consider an infant encountering red for the first time—no thought of “this is red,” only pure sensory impact.

5 Nishida (1990), founder of the Kyoto School and professor at Kyoto Imperial University, stands as one of Japan’s most significant modern philosophers. He conducted original philosophical work entirely in Japanese, establishing the systems of “logic of place” and “pure experience,” while also being hailed as one of the greatest Eastern philosophers of the 20th century.

6 Pre-reflective state: This perspective resembles Peirce’s Firstness (or even is Peirce’s Firstness), but Kojima embedded this starting point within the frameworks of “Mahayana emptiness” and “logic of place,” ultimately steering it toward entirely different directions. Kojima’s true originality and ‘Orientalism’ lie not in the Firstness of this “starting point,” but in the path taken after this starting point. Thus he does not merely replicate Western pragmatism, but rather reinterprets and transcends Western phenomenological insights through an Eastern (Zen, Hua Yen) ultimate framework, achieving genuine synthesis rather than simple transplantation.Nishida’s perspective ultimately points toward emptiness rather than infinite semiotics—this constitutes his fundamental divergence from Peirce and James, and his true contribution as an Eastern philosopher.

7 It must be emphasized that this study in no way posits that Western languages are “inherently more dualistic or deficient,” nor that Eastern languages are “inherently superior or non-dualistic.” From a pragmatic-communicative perspective, all natural languages are highly context-dependent in actual usage. Native English speakers also extensively employ ellipsis, implication, intonation, and body language to achieve non-explicit expression (Clark, 1996; Enfield and Levinson, 2019). Japanese’s topic-comment structure and subject omission merely grammaticalize certain pragmatic information, whereas Indo-European languages tend to leave such information at the pragmatic layer. This represents a division of labor rather than a hierarchy of superiority. However, within the framework of the weak Sapir-Wolf hypothesis, the degree of grammaticalization does yield systematic differences in default cognitive load: - When a cognitive operation (e.g., “Who is the agent?” or “Must subject and object be separated?”) is enforced into grammar, speakers find it harder to ‘ignore’ it during everyday thinking (Slobin, 1996‘s “thinking to speak”); - Japanese’s grammatical default focus on “topic/background” rather than “subject/agent” statistically facilitates native speakers’ unconscious prioritization of holistic-relational attention (Nisbett, 2003), thereby reducing the “grammatical resistance” to Nishida’s dialectical reconstruction in his native language. Therefore, the assertion that “Western languages tend to reinforce subject-object dualism” refers solely to the default cognitive tendency arising from grammatical default settings, not to superiority or inferiority in overall linguistic ability or culture. This tendency can be significantly weakened or reversed in proficient multilingual speakers (Athanasopoulos, 2011).

8 Absolute Contradictory Self-Identity (zettai mujunteki jiko dōitsu): Nishida’s most central ontological formula in his later period, meaning “two absolutely opposed terms, while mutually negating each other completely, are at a deeper level entirely the same thing.”For example: “I” and “the world” mutually negate each other absolutely (I am not the world, the world is not me), yet they are one and the same in the “place of absolute nothingness” (without “I,” there is no such thing as “the world”). See: Nishida (1987), Last Writings: Nothingness and the Religious Worldview.

9 Logic of Place (bashoteki ronri): True “being” must be encompassed and situated by a certain “nothingness.” This ultimate, all-encompassing ‘nothingness’ is the “Absolute Place of Nothingness.” As in: “A mirror reflects all things yet is itself reflected by none.” See: “From the Agent to the Perceiver” (1926–1928), included in The Collected Works of Kitaro Nishida, Volumes 4–5.

10 Relational Field: We can understand the “relational field” through Kitaro Nishida’s “logic of basho.” Nishida states: “The self is not an entity, but a place of opposites unified” (Nishida, 1987, p. 68). That is: the self, the world, and contradiction all emerge within a dynamic, symbiotic “field,” rather than existing independently.

11 Throughout this study, expressions such as “Western languages are more dualistic” and “Japanese is more context-sensitive” adopt the widely accepted meanings of “statistical tendencies” and “default processing biases” in cognitive linguistics and cultural psychology (Kay and Kempton, 1984; Lucy, 1992), rather than any linguistic or cultural essentialism or value judgments. We fully concur: from the perspective of practical communicative efficacy, no language is “superior” to others in terms of contextual dependency.
